# Personality Type Shapes Acute Anxiety Response to Music and Medicine Intervention During First Chemotherapy (PEGASUS-2)

**DOI:** 10.1007/s10484-025-09712-2

**Published:** 2025-05-10

**Authors:** Turan Karaoglu, Ozgur Tanriverdi

**Affiliations:** Faculty of Medicine, Department of Medical Oncology, Mugla Sıtkı Koçman University, Kötekli Mh. Marmaris Yolu Bulvarı No: 55 Menteşe, 48000 Mugla, Turkey

**Keywords:** Anxiety, Music and Medicine, Personality, Psycho-oncology, Stress

## Abstract

Music and medicine interventions are recognised for their effects on emotional regulation and stress reduction. However, limited research exists on how these interventions affect anxiety based on personality types, particularly in breast cancer patients undergoing chemotherapy. This study aimed to evaluate the effects of music and medicine interventions on anxiety levels according to personality types in women with breast cancer receiving adjuvant chemotherapy. In a controlled, cross-sectional case-control study, 120 women were randomly assigned to two groups: an intervention group (music and medicine + chemotherapy) and a control group (chemotherapy only). The music playlist included classical, folk, and pop genres, and participants’ anxiety levels were measured using the State-Trait Anxiety Inventory. Personality types were determined post-treatment using the Eysenck Personality Questionnaire - Revised Short Form. Serum C-reactive protein levels, blood pressure, and heart rate were also measured. Significant reductions were observed in anxiety levels, systolic blood pressure, heart rate, and serum C-reactive protein levels in the music intervention group compared to the control group. Neurotic individuals showed the most substantial improvement in anxiety, with reductions in systolic blood pressure and heart rate. Multivariate analysis revealed that both neurotic personality type and the music intervention were significant predictors of anxiety reduction. Music and medicine interventions provide notable benefits in reducing anxiety, particularly in neurotic individuals. Personalised music therapy based on personality types could enhance the quality of life for breast cancer patients undergoing chemotherapy. While this study focuses on the immediate effects of MMI during the first chemotherapy session, future research should explore the long-term impacts to better understand the sustained efficacy of such interventions in managing anxiety across multiple treatment cycles.

## Introduction

Music and medicine intervention (MMI) consciously uses music to regulate emotions, reduce stress, and enhance communication (Mao, 2022). It is widely used in psychiatric and psychosomatic conditions, taking advantage of music’s emotional and cognitive effects. Numerous studies highlight the positive effects of MMI on anxiety, providing not only short-term relief but also supporting long-term emotional balance and personal development. However, methodological variables such as music type, intervention timing, and individual preferences may lead to variation in study results (Bradt et al., 2021; Breaden Madden et al., 2023; Golden et al., 2021; Gustavson et al., 2021; Mao, 2022; Rossi et al., 2024).

Research has also expanded to explore the relationship between MMI and personality types (Dimitriadis et al., 2024; Feneberg et al., 2021; Rebecchini, 2021). Indeed, personality traits strongly influence music preferences, and studies have shown that MMI significantly influences personality traits (Park et al., 2013; Rossi et al., 2024). Personality types influence how people use music to develop emotional regulation strategies and often help individuals manage anxiety (Breaden Madden et al., 2023; Greenberg et al., 2015; Rossi et al., 2024). For example, extroverts often use music in social settings to boost energy, while introverts seek to relax with calmer music (Almusharraf & Almusharraf, 2021; Rossi et al., 2024). Neurotic individuals also tend to use music to manage negative emotions, while those with high psychoticism scores use music for expressive purposes (Lo et al., 2024; Mehnert-Theuerkauf et al., 2023; Rossi et al., 2024).

In cancer patients, anxiety prevalence varies by disease stage, type, and psychological characteristics. The connection between anxiety, cancer, and personality offers valuable insights into managing psychological conditions in cancer patients. Neurotic individuals often experience heightened anxiety, while extroverts tend to cope better with stress (Macía et al., 2020; Mitchell et al., 2011; Murnaghan et al., 2024).

While MMI have been widely recognized for their general stress–reducing properties, their specific application in managing chemotherapy-related symptoms remains underexplored. However, cancer patients undergoing chemotherapy often experience anxiety, fatigue, and stress as prominent side effects. Addressing these through relaxation techniques like MMI could provide targeted relief for these symptoms during the treatment trajectory.

To bridge this gap, our study focuses on the interplay between personality traits and the efficacy of MMI in alleviating anxiety during first chemotherapy session, grounded in a biopsychosocial framework. This study hypothesizes that MMI significantly reduce anxiety levels during first chemotherapy session, particularly neurotic individuals. The theoretical framework integrates personality psychology with the biopsychosocial model of cancer care, positing that individual differences in personality traits modulate the efficacy of therapeutic interventions like MMI.

Therefore, the study aims to evaluate the impact of MMI on anxiety in breast cancer patients undergoing chemotherapy, considering personality types, with a focus on physiological and inflammatory markers such as blood pressure (BP), heart rate (HR), and serum C- reactive protein (CRP) levels.

## Patients and Methods

### Study Design, Location, and Duration

This cross-sectional, observational case-control study aimed to evaluate the impact of MMI on anxiety in patients undergoing their first chemotherapy. The study group listened to a specially curated playlist during chemotherapy sessions, while the control group did not listen to any music. Anxiety levels, BP, heart rate, and serum CRP levels were compared between the two groups. The study was conducted in accordance with the Declaration of Helsinki. The Mugla Sitki Kocman University Scientific Research Ethics Committee approved the study (approval number 200123/122) on 2 May 2020. The study started on 10 June 2020, following the first COVID-19 lockdown. Conducted in a hospital chemotherapy unit, the study was paused from December 2020 to May 2021 due to the second lockdown, resuming in August 2021 and concluding in April 2023, with 120 patients enrolled.

## Study Population, Criteria, and Informed Consent

Participants were women diagnosed with breast cancer who had undergone surgery and were receiving adjuvant therapy with four cycles of anthracycline-cyclophosphamide every three weeks. Eligible patients, selected based on specific criteria, were informed about the study and gave their consent.

Inclusion criteria required histopathologically confirmed breast cancer, ages 18–70, and undergoing the first cycle of chemotherapy. Exclusion criteria included a history of psychiatric or cognitive illness, use of anxiolytic or antipsychotic treatment, head trauma, hearing problems, language comprehension issues, or local and systemic infections. Patients with prior experience in mindfulness-based cognitive therapy, meditation, yoga, psychotherapy, or MMI were also excluded. Depression was not specifically measured, but patients with a history of depression or recent antidepressant use were excluded to avoid its potential impact on the findings.

## Study Workflow

**Fig. 1:**
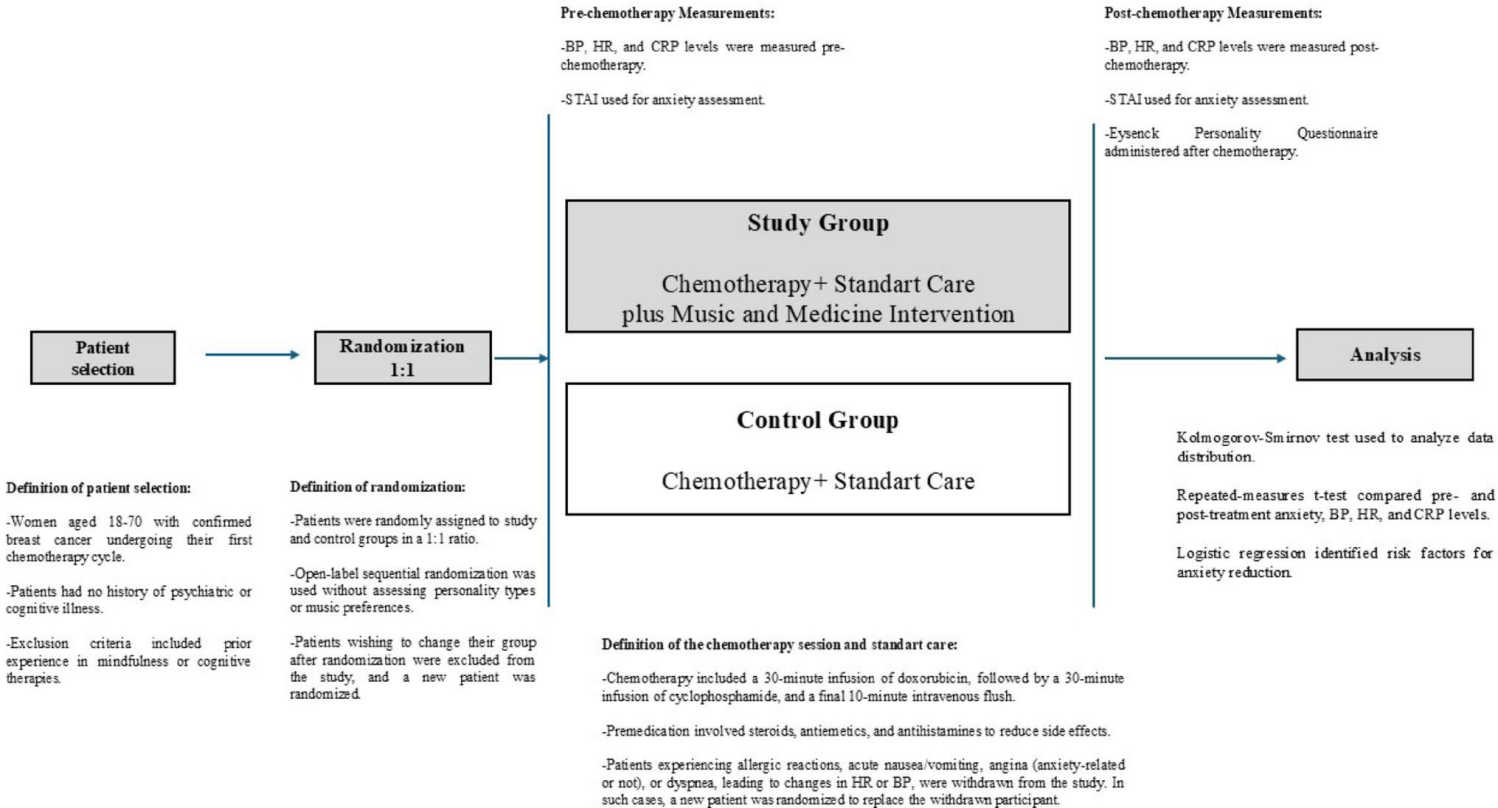
Study workflow.

Two groups were formed for the study (Fig. 1): the study group received standardised adjuvant chemotherapy along with an MMI for 60 min, excluding premedication and final flushing. The control group received the same chemotherapy without the MMI. Patients were randomly assigned to the groups in a 1:1 ratio using open-label sequential randomisation, without assessing personality types and music preference to avoid potential bias.

Upon arrival at the chemotherapy centre, patients were informed about the MMI. Those wishing to change their group were excluded from the study, and a new patient was randomised.

After consenting to treatment, all patients were seated and rested for 10 min in the chemotherapy unit. BP and HR were measured and recorded, the study scale was applied, intravenous access was established, and blood samples for CRP were taken.

For the study group, a predetermined music playlist was played through individual headphones starting with premedication until the chemotherapy session ended. The study group was treated in a separate, quiet room under the supervision of a researcher, and technical issues were monitored to avoid interruptions. The volume was set by the researcher to minimise discomfort. Headphones were removed only if the patient withdrew from the study, and after the session, the headphones were removed.

After chemotherapy, all patients rested for 10 min. Blood pressure and heart rates were measured again, the study scale was reapplied, and blood samples for CRP were taken. Subsequently, a personality type scale was administered. Patients without complications had their intravenous line removed and were discharged.

Patients who withdrew during the session, experienced technical issues preventing at least 10 min of music, had Grade 1 or higher allergic reactions, nausea, vomiting, angina, or acute dyspnoea requiring cessation of treatment were withdrawn from the study. A new patient was randomised to replace them.

All patients received standardised adjuvant chemotherapy with anthracycline-cyclophosphamide regimen. Premedication included steroids, granisetron antiemetics, and antihistamines, followed by a 30-minute infusion of doxorubicin, 30 min of cyclophosphamide, and a final 10-minute intravenous line flush.

## Music and Medicine Intervention

The pre-prepared sequential music playlist was applied for the 60-minute duration of the infusion of chemotherapeutic drugs, from the start of the infusion to its completion. The research team monitored the patients from a distance to ensure that the MMI continued, and no intervention was made except when there was uncertainty about whether the patients were listening to the music.

In this study, the music listening was conducted solely in the clinical research setting without any commercial purpose. Participants were not provided with any copies of the musical pieces, and the music was played solely during chemotherapy sessions through a listening list created on Spotify via a computer. The music used in the study was not commercially distributed, and no copyright infringement occurred.

The music playlist for the passive music and medicine Intervention included the following order: Mozart’s “Sonata for Two Pianos,” Beethoven’s “Moonlight Sonata,” Neşet Ertaş’s Turkish folk song “Divane Aşık Gibi,” Apocalyptica’s interpretation of “Nothing Else Matters,” Tarkan’s pop song “Kuzu Kuzu,” Mozart’s “Sonata for Two Pianos,” Beethoven’s “Moonlight Sonata,” instrumental Greek music, Neşet Ertaş’s “Divane Aşık Gibi” folk song, the instrumental version of the well-known Turkish folk song “Ey Gidi Karadeniz,” Mozart’s “Sonata for Two Pianos,” and Beethoven’s “Moonlight Sonata.”

When creating the music playlist, participants’ personal preferences were excluded to prevent bias, with various genres chosen to test the effects of different music styles on anxiety (Chen, 2023; Bowling, 2023; Özdemir et al., 2019; Theorell & Bojner Horwitz, 2019). The playlist primarily featured classical music, melancholic folk and slow-tempo rock, with an energetic pop song added for contrast. This pop song aimed to assess its impact compared to calmer genres. Pop music, while capable of boosting mood through its energizing effect, is also known to increase anxiety in some individuals. Therefore, it was consciously included in the playlist. The use of such contrasts is recommended in the literature to compare the physiological effects of different music genres (Bowling, 2023; Brandt et al., 2012; Nikolsky et al., 2020; Sezer, 2012; Theorell & Bojner Horwitz, 2019). The inclusion of slower, calming genres was intended to mitigate this effect.

Music combining classical rock and classical music with melancholic arrangements can provide a deep emotional experience and reduce anxiety. Turkish folk music, with nostalgic themes and natural instruments like the “bağlama,” “ney,” and “kaval,” offers relaxation and anxiety management. The literature suggests familiar music and natural sounds reduce stress more effectively than artificial ones (Brandt et al., 2012; Nikolsky et al., 2020; Sezer, 2012). Similar tones are found in Greek instrumental music (Karathanou et al., 2021). Cultural specificity in music selection adds another layer of complexity to the intervention, as familiarity with certain musical elements may amplify their calming effects. The inclusion of Turkish and Greek music aimed to explore the cultural resonance of familiar musical elements in reducing anxiety in our city, where localised on the South West coats in Türkiye. Additionally, classical music, particularly pieces by Mozart and Beethoven, is known for enhancing focus and reducing stress, making it a common choice for anxiety management. Works like Mozart’s “Sonata for Two Pianos” and Beethoven’s “Moonlight Sonata” are frequently used in studies on anxiety and pain due to their “regular rhythm, harmonic richness, and mid-low frequencies.” (Darki et al., 2022; Ding et al., 2023; Jenkins, 2011).

## Form and Scales Used in the Study

The case follow-up form included four sections. Section 1, completed at randomisation for both groups, gathered demographic and lifestyle information such as age, gender, occupation, education, marital status, economic level, and smoking habits. Among the demographic variables assessed, marital status and economic level were included to capture its potential impact on the psychological dynamics of cancer care. Marital status and economic level were included as they can influence the psychological resilience and social support available to cancer patients, which may, in turn, affect anxiety levels during first chemotherapy session.

Section 2 recorded BP, HR, and anxiety scale scores before and after treatment. Section 3 assessed personality type using a scale administered after a 10-minute resting period following chemotherapy. Section 4 recorded serum CRP results pre- and post-treatment.

The State-Trait Anxiety Inventory (STAI) was used to measure anxiety levels before and after chemotherapy. Developed by Spielberger et al. (1970), the STAI has two subscales with 20 questions each, measuring state (STAI-1) and trait (STAI-2) anxiety. The Turkish version has a reliability coefficient between 0.83 and 0.87 (Oner & Le Compte, 1998).

Personality traits were assessed using the Eysenck Personality Questionnaire - Revised Short Form (EPQ-RS) after chemotherapy (Eysenck et al., 1985). The EPQ-RS has 24 items across four dimensions: extraversion, neuroticism, psychoticism, and lie (Francis et al., 1992). The Turkish validation study confirmed four factors consistent with the original scale, with internal consistency coefficients of 0.78, 0.65, 0.42, and 0.64, and test-retest reliability of 0.84, 0.82, 0.69, and 0.69, respectively (Karanci et al., 2007).

### Measurement of CRP

Venous blood samples were collected from patients before and after chemotherapy to measure serum CRP levels, which are associated with inflammation and stress. CRP levels were analysed using nephelometric methods.

### Statistical Analysis

The distribution of variables was analysed using the Kolmogorov-Smirnov test. For variables that showed a normal distribution, means and standard deviations were presented, while for variables that did not follow a normal distribution, medians and minimum-maximum ranges were provided. Categorical data such as age, education level, occupation, marital status, economic status, place of residence, smoking habits, and personality type, which were quantitative variables with a normal distribution, were analysed using the chi-square test. The comparison of BP, HR, STAI-1 and STAI-2 scores, and serum CRP levels before and after treatment, as well as the changes between these measurements, were analysed using a repeated-measures t-test. The Kruskal-Wallis’s test was used to compare the differences in pre- and post-treatment changes based on personality types. Post-hoc analysis was performed. Finally, logistic (binary) regression analysis was conducted to identify independent risk factors influencing the reduction in anxiety. Statistical analysis was performed using the SPSS v25 programme and a p-value of < 0.05 was considered statistically significant.

## Results

The study group included 60 female patients diagnosed with breast cancer, and the control group also consisted of 60 female patients diagnosed with breast cancer. The average age of the patients in the study group was 45.8 ± 11.4 years, while in the control group it was 46.9 ± 10.9 years. No statistically significant difference in age was found between the two groups (*p* = 0.463).

Most of the patients in the study group were under 65 years old (*n* = 41, 68%), had undergraduate education (*n* = 25, 42%), were workers (*n* = 22, 37%), married (*n* = 36, 60%), lived in rural areas (*n* = 35, 58%), and had never smoked (*n* = 34, 57%). Similarly, many patients in the control group were under 65 years old (*n* = 44, 73%), had undergraduate education (*n* = 28, 47%), were workers (*n* = 24, 40%), married (*n* = 37, 62%), lived in rural areas (*n* = 36, 60%), and had never smoked (*n* = 37, 62%). No statistically significant differences were found between the study and control groups in terms of age, education level, occupation, marital status, economic status, place of residence, and smoking habits (Table 1).

**Table 1:** The demographical, clinical, and laboratory features of all patients included in the study

Variables	Study Group *n* = 60	Control Group *n* = 60	*P* value ^α^
Age; *n* (%) < 65 ≥ 65	41 (68) 19 (32)	44 (73) 16 (27)	0.139
Education; *n*(%)			
Literate Undergraduate Higher status	15 (25) 25 (42) 20 (33)	12 (20) 28 (47) 20 (33)	0.375
Occupational status			
Housewife Officer Worker	18 (30) 20 (33) 22 (37)	16 (27) 20 (33) 24 (40)	0.437
Marital status; n(%)			
Single Divorced Married	10 (17) 14 (23) 36 (60)	11 (18) 12 (20) 37 (62)	0.546
Economic status; n(%)			
Low input Middle input High input	16 (27) 20 (33) 24 (40)	18 (30) 20 (33) 22 (37)	0.296
Living place; n(%)			
Urban Rural	25 (42) 35 (58)	24 (40) 36 (60)	0.594
Smoking habits			
Never smoking Quit smoking (> 10 years) Habitual smoking	34 (57) 6 (10) 20 (33)	37 (62) 5 (8) 18 (30)	0.319
Personality type; n(%)			
Extravert Introvert Neurotic Psychotic	12 (20) 30 (50) 12 (20) 6 (10)	12 (20) 32 (53) 10 (17) 6 (10)	0.633

Statistical definitions: ^α^ Categorical data such as age, education level, occupation, marital status, economic status, place of residence, smoking habits, and personality type, which were quantitative variables with a normal distribution, were analysed using the chi-square test. A p-value of < 0.05 was considered statistically significant

Among the total 120 patients from both groups, 62 (52%) had an introverted personality type. This was followed by extroverted (*n* = 24, 20%), neurotic (*n* = 22, 18%), and psychotic (*n* = 12, 10%) personality types. In the study group, 50% of the patients were introverted, 20% extroverted, 20% neurotic, and 10% psychotic, while in the control group these rates were 53%, 20%, 17%, and 10%, respectively. No statistically significant difference was found in terms of personality type between the two groups (*p* = 0.633) (Table 1).

Post-treatment systolic BP showed a statistically significant reduction in the study group (9.74 ± 0.35 mmHg), while this reduction was more limited in the control group (4.09 ± 0.31 mmHg) (*p* < 0.0001). Regarding diastolic BP, no statistically significant difference was found between the two groups before and after treatment (*p* = 0.174). A statistically significant reduction in HR was observed in the study group (13.94 ± 3.96), while this change was smaller in the control group (7.56 ± 1.29) (*p* < 0.001). Additionally, statistically significant reductions in the STAI-1 and STAI-2 scores were found in the study group post-treatment (*p* < 0.0001 and *p* < 0.0001, respectively). A statistically notable decrease in serum CRP levels was also observed in the study group (*p* < 0.0001). The changes in clinical variables before and after treatment in both the study and control groups are shown in Table 2.

**Table 2:** Comparison of the study group and the control group in terms of pre-treatment and post-treatment values ​​as well as pre- and post-treatment changes in systolic blood pressure, diastolic blood pressure, anxiety scores and serum C-reactive protein levels

Variables	Study Group	Control Group	*P* value^α^
*n*	60	60	
Systolic blood pressure (mmHg)			
Pre-treatment	126.94 ± 1.37	125.33 ± 1.46	**< 0.0001**
Post-treatment	117.13 ± 1.09	121.45 ± 1.14	
Change score	9.74 ± 0.35	4.09 ± 0.31	
Diastolic blood pressure (mmHg)			
Pre-treatment	81.14 ± 1.42	82.09 ± 1.45	0.174
Post-treatment	76.94 ± 0.81	79.03 ± 1.11	
Change score	4.13 ± 0.19	3.04 ± 0.34	
Heart rate (Beat/minute)			
Pre-treatment	92.41 ± 8.74	92.34 ± 7.96	**< 0.001**
Post-treatment	79.38 ± 4.48	85.69 ± 6.31	
Change score	13.94 ± 3.96	7.56 ± 1.29	
STAI- 1			
Pre-treatment	46.4 ± 11.9	47.2 ± 12.4	**< 0.0001**
Post-treatment	39.6 ± 9.4	42.5 ± 10.9	
Change score	6.9 ± 2.5	4.8 ± 1.4	
STAI- 2			
Pre-treatment	39.42 ± 8.74	39.86 ± 9.05	**< 0.0001**
Post-treatment	37.33 ± 6.94	38.94 ± 8.96	
Change score	2.11 ± 1.81	0.93 ± 0.08	
Serum CRP level (mg/dl)			
Pre-treatment	5.32 ± 1.96	5.09 ± 1.93	**< 0.0001**
Post-treatment	3.74 ± 1.09	4.33 ± 1.48	
Change score	1.58 ± 1.92	0.78 ± 0.45	

STAI: The State-Trait Anxiety Inventory. CRP: C- reactive protein.

Statistical definitions: ^α^ The comparison of systolic and diastolic blood pressure, heart rate, STAI-1 and STAI-2 scores, and serum CRP levels before and after treatment, as well as the changes between these measurements, were analysed using a repeated-measures t-test. Statistical significance is indicated by p-values in bold. A p-value of < 0.05 was considered statistically significant.

Table 3 examines pre- and post-treatment changes according to personality types. Differences in systolic and diastolic BP, HR, STAI-1, STAI-2, and serum CRP levels were observed among extroverted, introverted, neurotic, and psychotic personality types. In the study group, neurotic individuals exhibited the greatest reduction in systolic BP (10.09 mmHg), and this reduction was statistically significant greater compared to the control group (*p* < 0.0001). Similarly, statistically significant reductions in HR and STAI-1 scores in neurotic individuals in the study group were more pronounced than in other personality types (*p* < 0.001 and *p* < 0.001, respectively).

**Table 3:** Comparison of the changes in systolic blood pressure, diastolic blood pressure, anxiety scores and serum C- reactive protein levels between pre-treatment and post-treatment in the study group and the control group according to personality type

Variables	Study Group (*n* = 60)				Control Group (*n* = 60)				*P* value^α^
Personality Type	Extravert	Introvert	Neurotics	Psychotic	Extravert	Introvert	Neurotic	Psychotic	
*n*	12	30	12	6	12	32	10	6	
Change SBP (mmHg)	9.33 (6.34–12.86)	8.34 (5.12–9.48)	10.09 (7.69–12.33)	7.52 (4.31–9.42)	3.07 (2.19–3.98)	4.31 (1.96–4.94)	3.11 (1.98–3.94)	3.86 (2.03–4.11)	**< 0.0001**
Change DBP (mmHg)	3.48 (1.16–4.04)	3.11 (1.17–3.96)	4.24 (2.31–4.86)	3.54 (1.34–3.86)	3.01 (0.98–3.41)	3.09 (1.07–3.46)	3.42 (1.96–4.16)	3.04 (0.59–3.84)	**0.016**
Change HR (Beat/minute)	11.4 (9.41–13.94)	10.86 (7.45–13.31)	14.09 (10.31–14.96)	8.94 (4.15–10.39)	8.41 (6.45–9.31)	7.41 (5.64–8.96)	6.98 (4.45–8.56)	7.04 (5.41–9.04)	**< 0.001**
Change STAI- 1	5.9 (5.1–6.6)	5.4 (4.7–6.1)	5.8 (5.2–7.8)	4.7 (2.9–6.1)	3.7 (2.8–4.2)	4.7 (2.9–5.1)	4.8 (3.1–5.1)	3.8 (3.1–4.6)	**< 0.001**
Change STAI- 2	1.6 (0.94–1.8)	1.8 (1.1–1.94)	2.33 (1.94–3.04)	1.4 (0.96–1.9)	0.86 (0.38–1.05)	0.89 (0.41–1.01)	0.91 (0.51–1.11)	0.89 (0.74–0.96)	**0.033**
Change serum CRP level (mg/dl)	1.74 (1.14–2.01)	1.49 (1.84–2.05)	1.94 (1.37–2.12)	1.37 (1.19–1.02)	0.86 (0.37–1.04)	0.91 (0.48–1.11)	0.81 (0.44–0.96)	0.89 (0.33–1.42)	**< 0.0001**

Abbreviations: SBP: Systolic blood pressure; DBP: Diastolic blood pressure; HR: Heart rate; STAI: The State-Trait Anxiety Inventory, CRP: C- reactive protein

Statistical definitions: ^α^ Comparison of the differences between pre-treatment and post-treatment according to personality types was done with the Kruskal-Wallis test due to the unequal distribution of the data. Post-hoc analysis was performed

Kruskal–Wallis H test statistics for comparisons among personality types were as follows: Change in SBP (H = 12.37), DBP (H = 8.94), HR (H = 13.94), STAI-1 (H = 14.37), STAI-2 (H = 9.33), and serum CRP levels (H = 16.96). All corresponding p-values are presented in the table. A p-value of < 0.05 was considered statistically significant. Statistical significance is indicated by p-values in bold

According to the univariate analysis results, age, occupation, economic status, personality type, and MMI were statistically significantly associated with the reduction in anxiety. In the multivariate analysis, neurotic personality type (OR: 2.46, 95% CI: 1.946–9.456, *p* < 0.0001) and MMI (OR: 2.96, 95% CI: 1.843–7.658, *p* < 0.0001) were identified as factors that statistically significantly influenced the reduction in anxiety. While the logistic regression model initially predicted anxiety levels with an accuracy of 59.5%, this rate increased to 89.4% with the developed model. Independent factors influencing the reduction in anxiety levels are shown in Table 4.

**Table 4:** Results of logistic regression analysis to determine the factors affecting the decrease in pre-treatment and post-treatment anxiety scores

Variables	Univariate analysis				Multivariate analysis			
	OR	95%CI Lower	95% CI Upper	*p* value	OR	95%CI Lower	95% CI Upper	*p* value
Age classification (≥ 65 year vs. <65 year)	**2.74**	**1.374**	**8.945**	**0.0011**	1.96	1.094	9.374	0.194
Educational level (Low education vs. High education)	1.94	0.345	9.485	0.475				
Occupation (Housewife vs. Other)	**2.62**	**1.564**	**7.945**	**< 0.001**	2.07	1.565	6.894	0.376
Economic status (Higher input vs. Other)	**3.06**	**1.546**	**11.456**	**< 0.0001**	2.37	0.948	5.645	0.546
Marital status (Single vs. Married)	2.45	0.746	4.567	0.642				
Personality type (Neurotic vs. Other)	**2.98**	**1.487**	**9.864**	**< 0.001**	**2.46**	**1.946**	**9.456**	**< 0.0001**
Music intervention (No vs. Yes)	**3.04**	**1.964**	**8.456**	**< 0.0001**	**2.96**	**1.843**	**7.658**	**< 0.0001**

Abbreviations: OR: Odds ratio; CI: confidence interval

Statistical Definitions: The variables in the table were included in the model because they were found to be significant in the univariate analysis. The variables that were not included in the model were not significant in the univariate analysis. Multivariate (binary) logistic regression analysis was performed because the data were not equally distributed. A P value less than 0.05 was considered statistically significant and shown in bold font. While the probability of predicting anxiety decrease was 59.5% at the beginning, this rate increased to 89.4% with the model created. The p value for the Omnibus test for the adequacy of the model was calculated as < 0.001. The Nagelkerke R squared value was 0.709.

## Discussion

This study found significant reductions in anxiety and physiological parameters in breast cancer patients following MMI, particularly among neurotic individuals. These findings align with previous research, which has shown the stress-relieving effects of music, especially in cancer patients (Bradt et al., 2021; Mehnert-Theuerkauf et al., 2023). Unlike previous studies, this research not only examines the general relaxing effects of music but also explores how specific personality traits, such as neuroticism, enhance or mitigate these effects. By focusing on physiological markers like CRP, this study provides objective evidence of MMI’s dual psychological and physiological benefits. The acute decrease in anxiety levels may shape patients’ perceptions of chemotherapy and their preparedness for subsequent sessions. Therefore, the sustainability of MMI’s effects in later sessions is as worthy of evaluation as its impact during the first session. While this study focuses on the immediate effects of MMI during the first chemotherapy session, future research should explore the long-term impacts to better understand the sustained efficacy of such interventions in managing anxiety across multiple treatment cycles.

Neuroticism is characterised by emotional instability and a higher propensity for anxiety (He et al., 2021; Sauer-Zavala et al., 2021; Widiger & Oltmanns, 2017). Neurotic individuals often experience more intense emotional reactions to stress, making them more responsive to interventions like music, which offers emotional regulation (He et al., 2021). The significant reductions in anxiety and physiological markers observed in neurotic patients in this study underscore the potential of MMI to provide relief for emotionally sensitive individuals.

In cancer patients, heightened sensitivity to stress can exacerbate anxiety, particularly in neurotic individuals (Bradt et al., 2021; Khan et al., 2018; Lima et al., 2020; Mehnert-Theuerkauf et al., 2023; Niedzwiedz et al., 2019; Tanriverdi et al., 2023). Personalised interventions that cater to individual personality traits may enhance the effectiveness of MMI. Neurotic individuals, who are more vulnerable to emotional fluctuations, may benefit the most from calming and emotionally resonant music (He et al., 2021; Widiger & Oltmanns, 2017). Tailoring MMI to align with personality types could optimise therapeutic outcomes, offering more personalised stress relief strategies.

The significant reduction in serum CRP levels observed in this study further supports the physiological benefits of MMI. Elevated CRP levels are associated with chronic stress and anxiety, both common in cancer patients (Khan et al., 2018). The decrease in CRP levels in neurotic individuals suggests that MMI may not only alleviate psychological stress but also reduce inflammation, offering broader health benefits (Niedzwiedz et al., 2019).

The large sample size of this study enhances the generalizability of its findings. The focus on individual differences in personality and music preferences distinguishes this study from others in the field of MMI for cancer patients. This is one of the few studies to include biochemical measurements, such as serum CRP levels, providing objective evidence of music’s physiological effects.

### Limitations

While the study provides valuable insights, it is not without limitations. The exclusion of personal music preferences may have impacted the results, as individual tastes play a crucial role in emotional responses to music. However, by using a diversified playlist, we aimed to minimise this bias. The exclusion of individual music preferences is a significant limitation of the study. Each individual has a unique emotional connection with music, and considering these preferences may further enhance the impact of the intervention. In this study, genre diversity was provided to balance this effect; however, it is important for future studies to incorporate designs that consider personalized music preferences.

Another limitation is the focus on short-term effects; long-term outcomes of music interventions were not evaluated. While the current study focuses on the short-term reduction of anxiety during the first chemotherapy cycle, this finding has potential implications for subsequent treatment cycles. Anxiety experienced during the initial cycle often influences patients’ perceptions and tolerance of chemotherapy in later cycles, potentially exacerbating chemotherapy side effects such as nausea, fatigue, and emotional distress. By mitigating acute anxiety early on, interventions like MMI may establish a positive psychological baseline, improving patient’s resilience and coping mechanisms for future cycles. Furthermore, studies have suggested that early reduction in stress and anxiety can have a cumulative effect, lessening the severity and frequency of side effects over time. Therefore, the observed short-term benefits of MMI may extend beyond the initial cycle, contributing to a more tolerable and effective chemotherapy experience.

Since the EPQ-RS personality test was administered after chemotherapy, the emotional effects of the music intervention or chemotherapy may have influenced participants’ responses. This is considered a potential limitation.

A history of depression and the use of antidepressants were exclusion criteria for participants; however, a formal depression screening tool was not used. This may limit the interpretation of the findings, especially due to the overlap of symptoms with neuroticism.

Additionally, the study only included female breast cancer patients, who limits the generalizability of the findings to other cancer types and male patients.

## Conclusion

This study highlights the efficacy of MMI in reducing anxiety and improving physiological outcomes in cancer patients, particularly those with neurotic personality traits. The findings suggest that MMI, when tailored to individual personality profiles, can offer a powerful tool for managing the psychological and physiological stress associated with cancer treatment. Personalised MMI could complement traditional cancer therapies, improving patient well-being and enhancing their ability to cope with the challenges of chemotherapy. Expanding on the immediate benefits identified in this study, future research should delve into the prolonged efficacy of MMI, particularly across diverse patient populations and extended treatment cycles. While this study focused on immediate effects, future research could investigate the long-term impact of repeated MMI sessions to provide a comprehensive understanding of its sustained efficacy.

## Data Availability

No datasets were generated or analysed during the current study.
